# Purpura annularis telangiectodes of Majocchi^☆^^☆☆^

**DOI:** 10.1016/j.abd.2020.02.007

**Published:** 2020-07-04

**Authors:** Aline Soares Garcez, Vitória Regina Pedreira de Almeida Rego, Thadeu Santos Silva

**Affiliations:** a Dermatology Service, Hospital Universitário Professor Edgard Santos, Universidade Federal da Bahia, Salvador, BA, Brazil; b Dermatology Service, Escola Bahiana de Medicina e Saúde Pública, Salvador, BA, Brazil

Dear Editor,

Purpura annularis telangiectodes of Majocchi is a rare subtype of pigmented purpuric dermatosis. It is more common in children and young females and predominantly affects the lower limbs, with symmetrical annular reddish-brown macules.^1^, ^2^ Little is known of its etiology, which may be associated with viral infections, chronic comorbidities, and use of medications. The diagnosis is clinical and histopathological. There is no consensus regarding treatment. Management is based on reports and case series, with variable response to the proposed treatments.^3^, ^4^

A 6-year-old female patient, daughter of consanguineous parents, presented lesions since 2 years old. The patient had no history of systemic symptoms, allergies, or continued use of medications, except sporadic use of paracetamol. In the beginning, the lesions were erythematous, and subsequently evolved to annular and/or irregular hyperchromic macules, symmetrical in the legs, as well as in the right upper limb, and with an isolated lesion in the anterior cervical region (Fig. 1). A skin biopsy of the right thigh was performed, demonstrating a lichenoid lymphohistiocytic infiltrate in the upper dermis and red blood cell extravasation, as well as foci of lymphocyte exocytosis and perivascular mononuclear infiltrate, without pigmentary incontinence or vasculitis (Fig. 2). Perls' Prussian blue staining indicated the presence of hemosiderin deposition in the papillary dermis (Fig. 3). The patient was screened for hematological, infectious, and rheumatological diseases, all negative. The authors opted for treatment with colchicine orally, with no response after five months of medication use.

**Figure 1:**
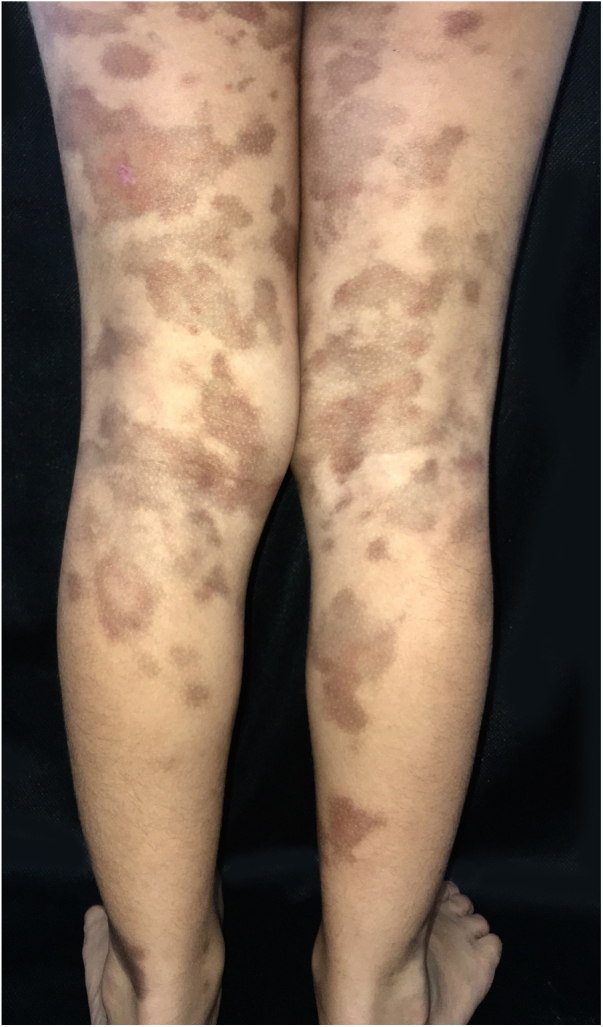
Multiple brownish macules on the legs and thighs.

**Figure 2:**
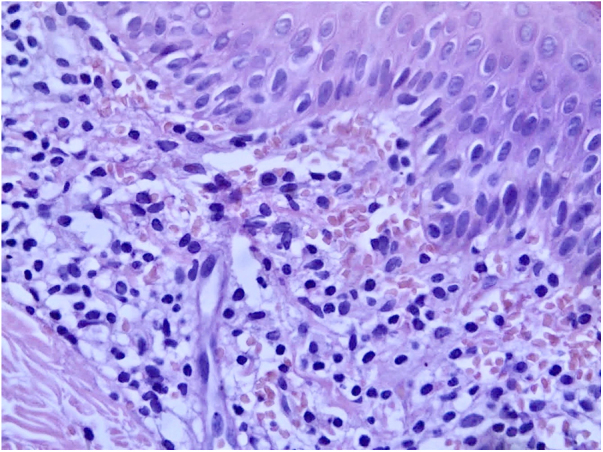
Lymphohistiocytic infiltrate in the upper dermis, as well as extravasation of red blood cells. It is possible to see some lymphocyte exocytosis (Hematoxylin & eosin, ×400).

**Figure 3:**
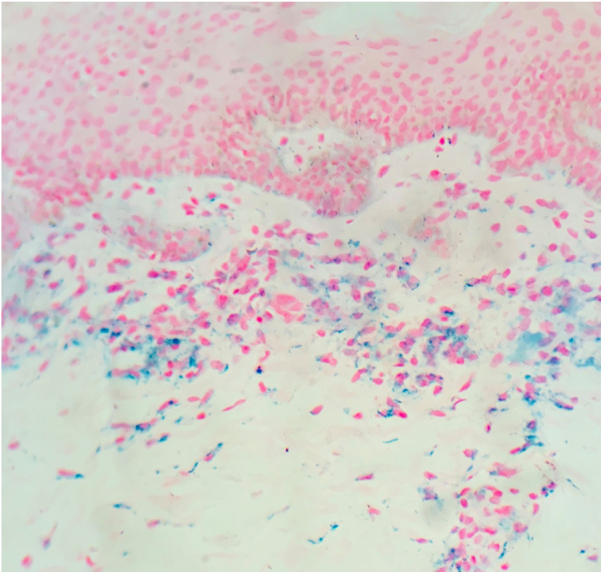
Presence of hemosiderin deposition in the papillary dermis (Perls' Prussian blue, ×200).

Purpura annularis telangiectodes of Majocchi is part of the pigmented purpuric dermatoses, which manifests as annular macules that are symmetrical, reddish-brown, and generally asymptomatic. It preferably affects children and young women, and there is no predominant ethnicity.^1^, ^2^, ^3^, ^4^ Commonly, it appears in the lower limbs; there appears to be an orthostatic component in the pathophysiology of the disease, as described in the present case. The etiology of pigmented purpuric dermatoses is not yet fully elucidated, and its triggers are not always detected; therefore, the etiology is idiopathic in most cases. An association with comorbidities such as diabetes mellitus, viral hepatitis, peripheral venous insufficiency, and use of certain medications has been reported, including the following: paracetamol, aspirin, carbamazepine, antihypertensives, infliximab, alpha-interferon, pseudoephedrine, raloxifene, and thiamine.^1^, ^4^ The present patient did not have any of the aforementioned comorbidities; the sporadic use of paracetamol was the only possible trigger identified in the clinical history. It is worth mentioning that most cases are idiopathic. The administration of paracetamol, even if occasional, may have been a trigger for the clinical picture as described by Kwon et al.^5^ The diagnosis of purpura annularis telangiectodes of Majocchi is clinical and histopathological. The clinical characteristics are closely related to the anatomopathological findings. Lesion pigmentation is due to the extravasation of red blood cells and deposition of hemosiderin seen in the papillary dermis. It is also possible to observe perivascular inflammatory infiltrate composed of lymphocytes, histiocytes, and Langerhans cells. These findings corroborate the possible association of pigmented purpuric dermatoses with cellular immunity, contributing to capillary fragility. Humoral immunity also appears to play a role, which can be evidenced by direct immunofluorescence, which can detect perivascular deposits of immunoglobulins and complement. Foci of lymphocyte exocytosis may occur, as observed in this case. Leukocytoclastic vasculitis or epidermotropism are not observed.^3^, ^4^ The knowledge of this entity and its early diagnosis can allow the assessment of the presence of triggers and guide its management.

## Financial support

None declared.

## Authors’ contributions

Aline Soares Garcez:; elaboration and writing of the manuscript; critical review of the literature.

Vitória Regina Pedreira de Almeida Rego: Approval of the final version of the manuscript; critical review of the literature.

Thadeu Santos Silva: Approval of the final version of the manuscript; elaboration and writing of the manuscript; intellectual participation in propaedeutic and/or therapeutic conduct of studied cases; critical review of the literature, critical review of the manuscript.

## Conflicts of interest

None declared.
